# Modeling *APOE*, morbidity, and mortality: a reproducibility challenge for genetic epidemiology

**DOI:** 10.3389/fgene.2026.1782955

**Published:** 2026-02-20

**Authors:** Erling Häggström Gunfridsson

**Affiliations:** Centre for Demographic and Ageing Research (CEDAR), Umeå University, Umeå, Sweden

**Keywords:** *APOE*, causal inference, covariate adjustment, genetic epidemiology, mortality, pleiotropy, population structure, reproducibility

## Abstract

APOE is among the most extensively studied genetic loci in research on aging, morbidity, and mortality. Despite its well-established biological roles, empirical findings on the association between *APOE* and mortality remain inconsistent across studies. This heterogeneity is often attributed to biological complexity. In this Perspective, we argue that much of the apparent inconsistency instead reflects differences in modeling choices, variable definitions, and reporting practices, resulting in limited reproducibility and comparability. We highlight how pleiotropy, age-dependent effects, and selective survival make *APOE* particularly sensitive to analytical decisions. We focus on three underappreciated sources of irreproducibility: selective exclusion of rare *APOE* genotypes, lack of standardized baseline models, and routine adjustment for variables that are not confounders under Mendelian inheritance. We argue that all observed *APOE* genotypes should be included in primary analyses, that parsimonious baseline models adjusted only for variables independent of genotype should always be reported, and that overadjustment can obscure rather than clarify genetic effects. We propose a set of conceptual principles to improve reproducibility in studies of *APOE*, morbidity, and mortality, with implications for genetic epidemiology more broadly.

## 1 Introduction

*APOE* plays a central role in research on lipids and cardiovascular disease, neurodegenerative disorders such as Alzheimer’s disease, and aging and survival more broadly (Corder et al., 1993; Deelen et al., 2011). Despite this extensive literature, studies of *APOE* and mortality have produced heterogeneous results across studies, with reports of protective, harmful, or null associations depending on study design and population (Wolters et al., 2019). An important contributor to this heterogeneity is the strongly age-dependent nature of *APOE* effects. For several key outcomes, including cognition, cognitive decline, and Alzheimer’s disease risk, associations with *APOE* at younger ages are often weak, inconsistent, or directionally heterogeneous, with evidence suggesting antagonist pleiotropy or context-dependent effects rather than uniform risk (Weissberger et al., 2018; Remer et al., 2020; Reynolds et al., 2019; Chang et al., 2016). Associations tend to increase markedly in later adulthood and may attenuate again at very old ages. Differences in age at study entry, follow-up windows, and choice of time scale can therefore generate divergent estimates even under otherwise similar analytical strategies.

This heterogeneity is often attributed to biological complexity, pleiotropy, or context-dependent effects. While these explanations are plausible, they obscure an equally important source of inconsistency: differences in analytical choices. We argue that associations between *APOE* and mortality are highly sensitive to modeling decisions, making reproducibility, rather than biological uncertainty, the central challenge.

This issue has important implications for meta-analyses, interpretation of genetic risk factors, and the generalizability of findings in genetic epidemiology. Without transparent and comparable analytical frameworks, it becomes difficult to distinguish genuine biological differences from artifacts of study design and modeling (Ioannidis, 2008).

These issues mirror broader concerns about reproducibility in biomedical research, where analytic flexibility and heterogeneous reporting practices have been identified as key contributors to irreproducible findings. Studies of *APOE* and mortality thus exemplify how the replication crisis in genetic epidemiology is driven not only by limited power or biological complexity, but by avoidable differences in modeling and reporting.

## 2 Variable definition and reporting practices

A substantial source of irreproducibility in studies of *APOE*, morbidity, and mortality arises not from biology, but from inconsistent variable definitions and reporting practices. Even when similar cohorts and outcomes are analyzed, differences in how *APOE* genotypes are handled, which covariates are included, and which models are reported can lead to results that are not meaningfully comparable across studies (Hernán and Robins, 2016).

### 2.1 Definition and inclusion of *APOE* genotypes

*APOE* is typically defined by the ε2/ε3/ε4 haplotypes derived from two coding variants. Despite this apparent simplicity, there is considerable heterogeneity in how *APOE* is operationalized in empirical studies. Common approaches include ε4 carrier status, ε4 allele count, or selective inclusion of only the most frequent genotypes.

We argue that all observed *APOE* genotypes should be included in primary analyses, even when some categories are rare and individually non-significant. Excluding genotypes such as ε2ε2 or ε2ε4 due to limited statistical power conflates absence of evidence with evidence of absence and introduces avoidable bias (Sterne and Davey Smith, 2001; Ioannidis, 2008). Such exclusions are often data-driven rather than design-based and can distort effect estimates for the remaining genotype categories.

Importantly, rare genotype categories contribute valuable information in a cumulative evidence framework. While a single study may provide imprecise estimates for these groups, inclusion enables synthesis across studies through meta-analysis, where small contributions can aggregate into meaningful inference (Evangelou and Ioannidis, 2013). Selective omission of genotypes undermines this process and reduces the interpretability of between-study comparisons.

For transparency and reproducibility, studies should explicitly report genotype frequencies, coding schemes, any pooling or exclusion decisions, and results from models including all observed *APOE* genotypes.

### 2.2 Covariate selection and baseline models

A second major barrier to reproducibility is the lack of standardized baseline models. Studies of *APOE* and mortality frequently differ in their choice of covariates, with some including extensive adjustment for socioeconomic, behavioral, and clinical variables, while others adopt more parsimonious approaches. As a result, reported effect estimates often reflect different estimands rather than conflicting evidence (Greenland et al., 1999; Westreich and Greenland, 2013).

We propose that simple baseline models should always be reported, adjusted only for variables known to be independent of *APOE* genotype. Such models typically include age, sex, and ancestry-related variables, such as self-reported ethnicity, country of birth, or genetic principal components when available, and serve as a reproducibility anchor across studies. In studies lacking detailed ancestry information, this limitation should be stated explicitly, and baseline estimates interpreted as potentially conflating genetic effects with unmeasured population structure rather than compensated for by adjustment for downstream social variables.

More elaborate models incorporating behavioral, socioeconomic, or clinical variables may be appropriate for specific research questions, particularly those addressing mediation or effect modification. However, without a common baseline model, it becomes impossible to disentangle true biological differences from differences in analytical choices (Hernán and Robins, 2016; VanderWeele, 2015).

### 2.3 Mendelian principles and overadjustment

Under Mendelian inheritance, *APOE* genotype is assigned at conception and is therefore independent of post-conceptional social and behavioral factors (Smith and Ebrahim, 2004; Lawlor et al., 2008). Accordingly, post-conceptional variables such as socioeconomic status, education, or adult lifestyle do not function as confounders of genetic effects in the causal sense, although they may act as mediators or effect modifiers and are therefore relevant for specific research questions.

Consequently, adjustment for such variables is not required in baseline genetic models and may, in some cases, be counterproductive. Socioeconomic factors may lie downstream of health processes influenced by *APOE* or act as colliders through selection mechanisms, particularly in older cohorts (Schisterman et al., 2009; Cole et al., 2010). Routine adjustment for these variables risks introducing bias while simultaneously reducing comparability across studies.

This does not imply that social or environmental factors are irrelevant. Rather, their role should be addressed explicitly, through stratified analyses, interaction models, or mediation frameworks, rather than implicitly absorbed into baseline adjustment sets. In the specific case of *APOE* ε4, lifestyle variables are frequently examined as potentially modifiable pathways or interaction partners (Deza-Lougovski et al., 2024), which further underscores the importance of distinguishing baseline genetic models from analyses aimed at mediation or interaction.

Observed associations between *APOE* genotype and socioeconomic variables in stratified populations typically reflect underlying population structure rather than causal effects of socioeconomic status on genotype; appropriate adjustment should therefore target ancestry or population structure, not downstream social variables.

## 3 Discussion

Although this Perspective is motivated by the extensive literature on *APOE*, morbidity, and mortality, the issues raised are not specific to this locus. Rather, *APOE* illustrates a broader challenge in genetic epidemiology: how modeling choices, variable definitions, and reporting practices shape reproducibility and cumulative inference.

A central implication is that irreproducibility often reflects differences in estimands rather than contradictory evidence. Studies that differ in genotype inclusion, covariate adjustment, time scales, or outcome definitions may address fundamentally different questions, even when nominally investigating the same association (Greenland et al., 1999; Westreich and Greenland, 2013). Although much of the contemporary *APOE* literature now converges on broadly similar baseline specifications, the persistence of heterogeneous modeling choices even in this comparatively mature field illustrates how sensitive genetic associations remain to analytical decisions.

These considerations are increasingly relevant as genetic epidemiology moves toward larger consortia, meta-analyses, and polygenic approaches. Polygenic scores aggregate effects across many loci, many of which are pleiotropic and subject to similar modeling sensitivities as *APOE* (Wray et al., 2014; Boyle et al., 2017; Mostafavi et al., 2020). If reproducibility is compromised at the level of single, well-characterized loci, it is unlikely to improve when complexity is scaled up.

More broadly, concerns about reproducibility extend beyond genetic epidemiology and reflect structural challenges in modern biomedical research (Ioannidis, 2005; Peng, 2011; Munafò et al., 2017). In this sense, the challenges illustrated by *APOE*–mortality studies are not exceptional, but representative of the broader replication crisis in genetic epidemiology, where flexible modeling choices and inconsistent reporting continue to undermine cumulative inference.

In conclusion, *APOE* serves as a stress test for genetic epidemiology. Its well-established biology, strong effects on multiple disease pathways, and sensitivity to selection and modeling decisions expose weaknesses in current analytical practice. Addressing these weaknesses requires that baseline models prioritize reproducibility, transparency, and comparability over statistical sophistication within individual studies. Concretely, improving reproducibility in studies of *APOE* and mortality requires that all observed *APOE* genotypes be included in primary analyses and that simple baseline models, adjusted only for age, sex, and ancestry-related variables used to account for population structure, be routinely reported alongside more complex specifications.

## Data Availability

The original contributions presented in the study are included in the article/supplementary material, further inquiries can be directed to the corresponding author.
